# A SYSTEMATIC REVIEW OF EFFECTIVE STRATEGIES OR INTERVENTIONS TO SUPPORT DRIVING CESSATION AMONG OLDER ADULTS

**DOI:** 10.1093/geroni/igad104.2826

**Published:** 2023-12-21

**Authors:** Anne Dickerson, Tadhg Stapleton, Isabel Margot-Cattin, Priscilla Harries, Isabelle Gélinas, Moon Choi, Lizette Swanepoel, Carolyn Unsworth

**Affiliations:** East Carolina University, Greenville, North Carolina, United States; Trinity College Dublin, Dublin, Dublin, Ireland; UNiversity of Applied Sciences of Western Switzerland (HES-SO), Lausanne, Vaud, Switzerland; Kingston University, Kingston Upon Thames, England, United Kingdom; McGill University, Montreal, Quebec, Canada; KAIST, Daejeon, Republic of Korea; Stellenbosch University, Cape Town, Western Cape, South Africa; Federation University, Churchill, Victoria, Australia

## Abstract

Driving remains the primary mode of transportation and often the only mode for rural and suburban areas. However, research is clear we all outlive our ability to drive. Nevertheless, as community mobility is essential to health and quality of life, we need to study and invest in understanding how to use and learn alternative means of transportation, especially for the vulnerable aging. A systematic review was designed to examine the literature for evidence-based strategies or interventions to support driving cessation among older adults while maintaining community mobility. The team of 13 international researchers from seven countries used the Covidence software with support of an expert health sciences librarian. Using multiple search terms (e.g., driving retirement; driving cessation; driving transition; interventions; strategies; older adults), 7317 studies were imported for screening with 7059 eliminated; 205 full texts were assessed for eligibility by 2-3 members. With 187 excluded, 19 studies were included and examined by the two primary authors. Results demonstrate low evidence of effectiveness of programmatic interventions, although multiple strategies have been identified. Overwhelmingly, the evidence supports starting very early with discussions about the process of transitioning from driving to being a passenger; particularly true with progressive diagnoses such as dementia. This presentation will highlight the programs currently available, strategies that appear to be successful, and review current toolkits for supporting driving cessation as well as emerging interventions and strategies.

